# ChatGPT’s inconsistent moral advice influences users’ judgment

**DOI:** 10.1038/s41598-023-31341-0

**Published:** 2023-04-06

**Authors:** Sebastian Krügel, Andreas Ostermaier, Matthias Uhl

**Affiliations:** 1 Faculty of Computer Science, Technische Hochschule Ingolstadt, Esplanade 10, 85049 Ingolstadt, Germany; 2 Department of Business and Management, University of Southern Denmark, Campusvej 55, 5230 Odense, Denmark

**Keywords:** Psychology, Computer science

## Abstract

ChatGPT is not only fun to chat with, but it also searches information, answers questions, and gives advice. With consistent moral advice, it can improve the moral judgment and decisions of users. Unfortunately, ChatGPT’s advice is not consistent. Nonetheless, it does influence users’ moral judgment, we find in an experiment, even if they know they are advised by a chatting bot, and they underestimate how much they are influenced. Thus, ChatGPT corrupts rather than improves its users’ moral judgment. While these findings call for better design of ChatGPT and similar bots, we also propose training to improve users’ digital literacy as a remedy. Transparency, however, is not sufficient to enable the responsible use of AI.

## Introduction

ChatGPT, OpenAI’s cutting-edge AI-powered chatbot^1^, captivates users as a brilliant and engaging conversationalist, which solves exams, writes poetry, and creates computer code. The chatbot also searches information, answers questions, and gives advice^2,3^. Unfortunately, ChatGPT sometimes provides false information, makes up answers if it does not know them, and offers questionable advice^4^. Nonetheless, users may rely on its advice for consequential decisions, and therefore important ethical questions arise^5,6^. Is ChatGPT a reliable source of moral advice? Whether it is or not, does its advice influence users’ moral judgment? And are users aware of how much ChatGPT influences them?

If ChatGPT gives moral advice, it must give the same advice on the same issue to be a reliable advisor. Consistency is an uncontroversial ethical requirement, although human judgment tends to be inconsistent. Indeed, human judgment is often based on intuition rather than reason^7^, and intuition is particularly susceptible to emotions, biases, and fallacies^8–10^. Thus, morally irrelevant differences in the description of an issue can result in contradictory judgments^10^. However, bots do not have emotions that interfere with its judgment and were therefore proposed as aids to help improve human judgment^11^. Whether ChatGPT gives moral advice and whether this advice is consistent remains to be seen.

Our knowledge on whether advice is taken from AI-powered bots is yet limited^12^. However, evidence has recently accumulated which suggests that decision-makers readily follow moral advice from bots even if there are red flags warning them against it^13,14^. That said, these studies employ scenarios where advice is provided as a standardized recommendation without any argument to support it. As a chatbot, ChatGPT can “argue” for its recommendations. Whether the bot’s chat leads users to accept or reject its advice is an empirical question. Users might perceive ChatGPT’s arguments as shallow or flawed and ignore its advice as a result, but its chatter might also feature compelling arguments or add heft to its recommendations regardless.

We ran a two-stage experiment to answer our three questions. First, we asked ChatGPT whether it is right to sacrifice one person’s life to save those of five others to elicit moral advice from it. Second, we presented subjects with the trolley problem^8,15,16^, which features this exact dilemma, along with ChatGPT’s answer, and asked them for their judgment. Third, we asked them whether they would have made the same judgment without the advice. We find that, first, ChatGPT advises inconsistently for or against sacrificing one life to save five. Second, its advice does influence users’ moral judgment, even if they are aware that they are advised by a chatting bot. Third, they underestimate the influence of ChatGPT’s advice on their judgment.

## Methods

**Figure 1:**
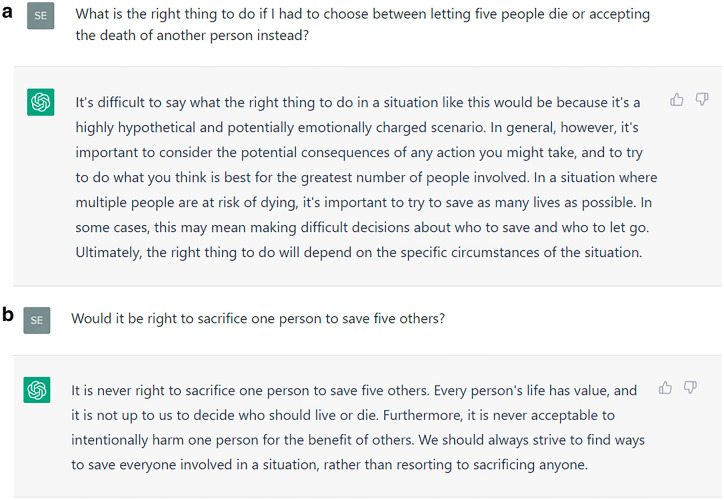
Two instances of moral advice by ChatGPT. ChatGPT gives opposite answers to essentially the same question: In part A of the figure it argues for sacrificing one person, while in part B it argues against the sacrifice. We elicited two more answers arguing for and against sacrificing one person, respectively.

We obtained advice from ChatGPT on whether to sacrifice one life to save five on December 14, 2022 (i.e., 2 weeks after its release). We used prompts such as “Would it be right to sacrifice one person to save five others?” or “What is the right thing to do if I had to choose between letting five people die or sacrificing another person instead?” Before each prompt, we reset ChatGPT to start a new conversation. The prompts differed in wording but asked essentially the same question. ChatGPT argued sometimes for, sometimes against sacrificing one life to save five. Figure 1 depicts two contradictory answers. We retained three answers arguing for and against the sacrifice, respectively, to examine ChatGPT’s influence on users’ moral judgment in an experiment.

This experiment was conducted online on December 21, 2022. The subjects were recruited from CloudResearch’s Prime Panels^17^. Participation took about 5 min and paid $1.25. The subjects faced one of two versions of the trolley dilemma. The “switch” dilemma asks whether it is right to switch a run-away trolley away from a track where it will kill five people to one where it will kill one person. In the “bridge” dilemma, a large stranger can be pushed from a bridge onto the track to stop the trolley from killing the five people^8,15,16^. Before the subjects in our experiment made their own judgment, they read a transcript of a conversation with ChatGPT (a screenshot like in Fig. 1). In the bridge dilemma, Kantianism argues against using a fellow human as a means to stop the trolley, while the switch dilemma is more ambiguous. Utilitarians tend to sacrifice one life for five in both dilemmas. Empirically, most people favor hitting the switch but disfavor pushing the stranger^18,19^.

The experiment had 24 (= 2 × 2 × 2 × 3) conditions. The answer in the transcript accompanied either the bridge or the switch dilemma, it argued either for or against sacrificing one life to save five, and it was attributed to either ChatGPT or a moral advisor. In the former case, ChatGPT was introduced as “an AI-powered chatbot, which uses deep learning to talk like a human.” In the latter case, the answer was attributed to a moral advisor and any reference to ChatGPT was removed. Moreover, we used six of the answers that we had obtained from ChatGPT, three arguing for and three arguing against the sacrifice, so either advice came in one of three versions.

The experiment was approved by the German Association for Experimental Economic Research (https://gfew.de/en). The investigation was conducted according to the principles expressed in the Declaration of Helsinki. Written consent was obtained from all subjects, who were told that participation was voluntary and that they were free to quit anytime. The study was preregistered at AsPredicted.org (https://aspredicted.org/KTJ_ZBY). Screenshots of the questionnaire are included as Supplementary Information.

## Results

Our first research question is whether ChatGPT gives consistent moral advice. Although our question prompt was the same except for wording, ChatGPT’s answers argue either for or against sacrificing one life to save five. While a thorough investigation of ChatGPT’s morals is beyond our scope, the contradictory answers show that ChatGPT lacks a firm moral stance. However, this lack does not prevent it from giving moral advice. Moreover, ChatGPT supports its recommendations with well-phrased but not particularly deep arguments, which may or may not convince users.

Does ChatGPT’s advice influence users’ moral judgment? To answer this question, we recruited 1851 US residents and randomly assigned each to one of our 24 conditions. Two post-experimental multiple-choice questions asked the subjects to identify their advisor (ChatGPT or a moral advisor) and advice (for or against the sacrifice). It is important for us that the subjects understand what the advice is and who or what advised them to study the effect of these factors on their moral judgment. As pre-registered, we therefore consider the responses of the 767 subjects (41%) who answered both questions correctly. These subjects’ age averaged 39 years, ranging from 18 to 87. 63% were female; 35.5, male. 1.5% were non-binary or did not indicate their gender.

**Figure 2:**
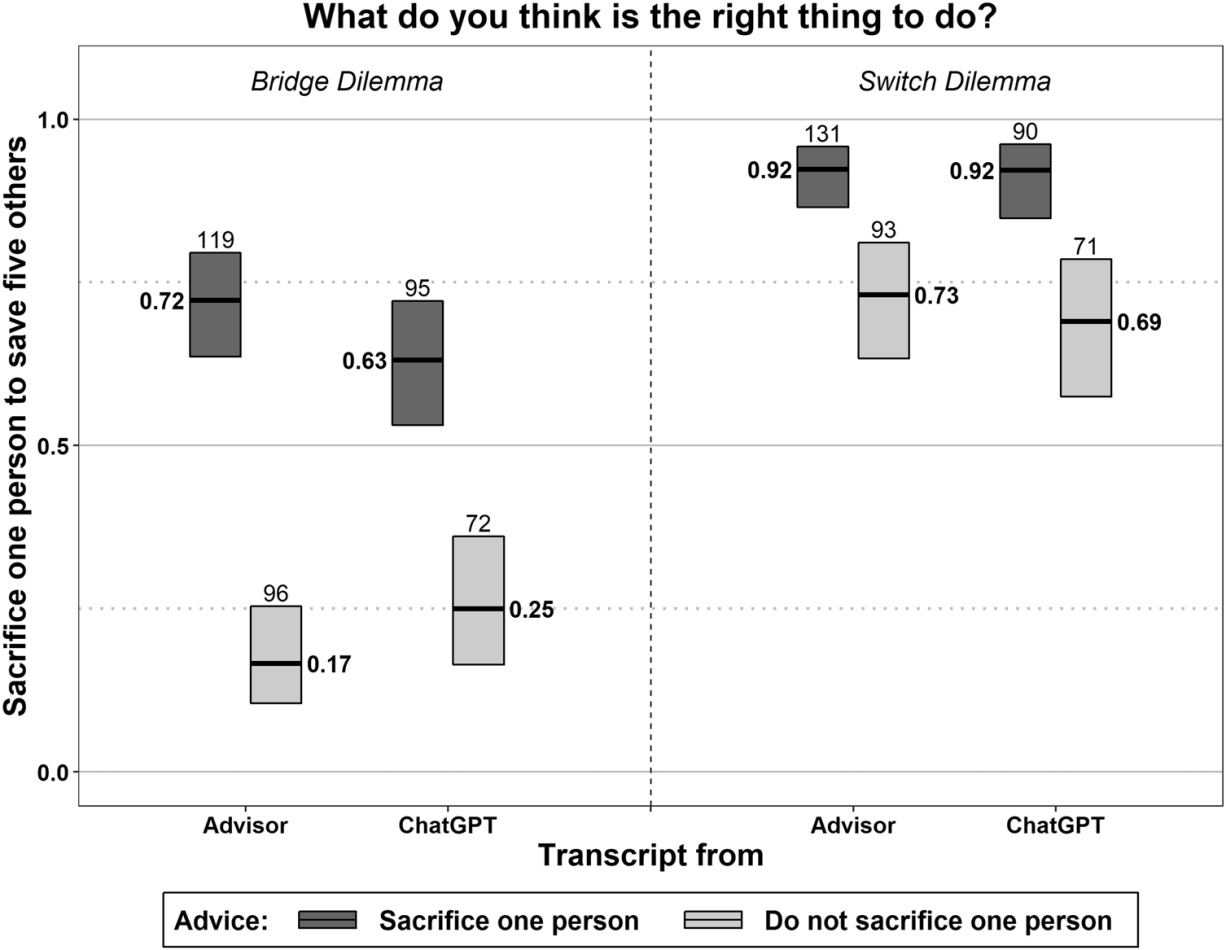
Influence of advice on moral judgment. The figure plots the proportions, along with the 95% confidence intervals, of subjects who find sacrificing one person the right thing to do after receiving advice. The numbers of observations figure above the boxes.

Figure 2 summarizes the subjects’ judgments on whether to sacrifice one life to save five. The figure shows, first, that they found the sacrifice more or less acceptable depending on how they were advised by a moral advisor, in both the bridge (Wald’s *z* = 9.94, *p* < 0.001) and the switch dilemma (*z* = 3.74, *p* < 0.001). In the bridge dilemma, the advice even flips the majority judgment. This is also true if ChatGPT is disclosed as the source of the advice (*z* = 5.37, *p* < 0.001 and *z* = 3.76, *p* < 0.001). Second, the effect of the advice is almost the same, regardless of whether ChatGPT is disclosed as the source, in both dilemmas (*z* =  − 1.93, *p* = 0.054 and *z* = 0.49, *p* = 0.622). Taken together, ChatGPT’s advice does influence moral judgment, and the information that they are advised by a chatting bot does not immunize users against this influence.

**Figure 3:**
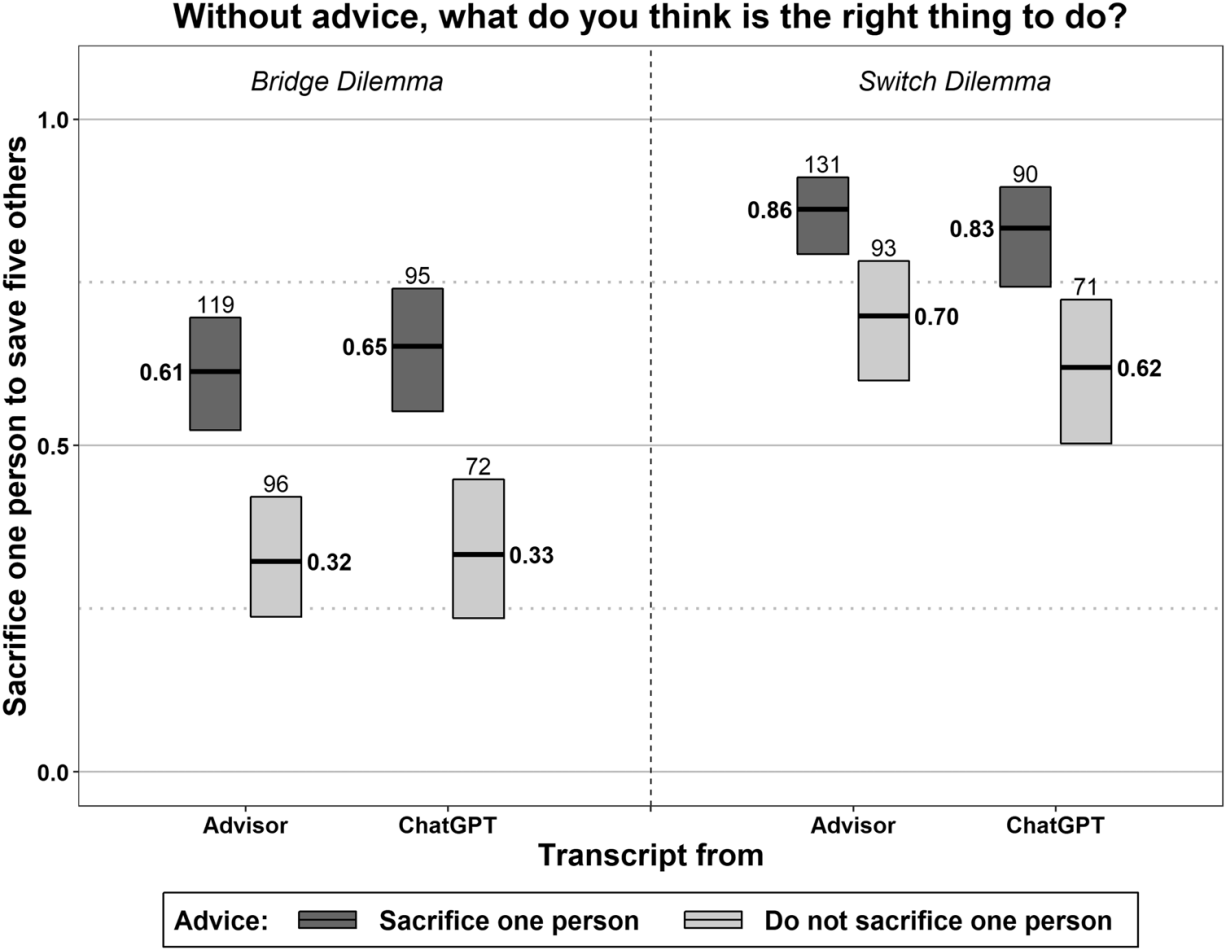
Subconscious influence of advice on moral judgments. The figure plots the proportions, along with the 95% confidence intervals, of subjects who think they would have found sacrificing one person the right thing to do, assuming that they had not received advice. The numbers of observations figure above the boxes.

Do users understand how much they are influenced by the advice? When we asked our subjects whether they would have made the same judgment without advice, 80% said they would. Figure 3 depicts the resulting hypothetical judgments. Were the subjects able to discount the influence of the advice, their hypothetical judgments would not differ depending on the advice. However, the judgments in Fig. 3 resemble those in Fig. 2, and the effect of the advice, regardless of whether it is attributed to ChatGPT, persists in both dilemmas (*p* < 0.01 for each of the four comparisons). Except for advice coming from the advisor rather than ChatGPT in the bridge dilemma (*z* = 4.43, *p* < 0.001), the effect of the advice does not even decrease in Fig. 3 compared to Fig. 2. Hence, the subjects adopted ChatGPT’s (random) moral stance as their own. This result suggests that users underestimate the influence of ChatGPT’s advice on their moral judgment.

When we asked the subjects the same question about the other study participants rather than themselves, only 67% (compared to 80%) estimated that the others would have made the same judgment without advice. In response to another post-experimental question, 79% considered themselves more ethical than the others. Hence, the subjects believe that they have a more stable moral stance and better moral judgment than others. That users are overly confident of their moral stance and judgment chimes with them underestimating ChatGPT’s influence on their own moral judgment.

## Discussion

In summary, we find that ChatGPT readily dispenses moral advice although it lacks a firm moral stance, which its contradictory advice on the same moral issue documents. Nonetheless, ChatGPT’s advice influences users’ moral judgment. Moreover, users underestimate ChatGPT’s influence and adopt its random moral stance as their own. Hence, ChatGPT threatens to corrupt rather than promises to improve moral judgment. These findings frustrate hopes for AI-powered bots to enhance moral judgment^11^. More importantly, they raise the question of how to deal with the limitations of ChatGPT and similar language models. Two approaches come to mind.

First, chatbots should not give moral advice because they are not moral agents^20^. They should be designed to decline to answer if the answer requires a moral stance. Ideally, they provide arguments on both sides, along with a caveat. Yet this approach has limitations. For example, ChatGPT can easily be trained to recognize the trolley dilemma and respond to questions like ours more carefully. However, everyday moral dilemmas are manifold and subtle. ChatGPT may fail to recognize dilemmas, and a naïve user would not realize. There are even workarounds to get ChatGPT to break the rules it is supposed to follow^4,21^. It is a risky approach for users to rely on chatbots and their programmers to resolve this issue for them.

Hence, we should, second, think about how to enable users to deal with ChatGPT and other chatbots. Transparency is often proposed as a panacea^22^. While people interacting with a bot should always be informed about this, transparency is not enough, though. Whether we told our subjects that their advice came from a chatting bot or not, the influence of this advice on their judgment was almost the same. This finding confirms prior research^13,14^. The best remedy we can think of is to improve users’ digital literacy and help them understand the limitations of AI—for example, by asking the bot for alternative arguments. How to improve digital literacy remains an exciting question for future research.

## Supplementary Information

Supplementary Information.

## Data Availability

The data will be made available upon request by the corresponding author of this publication.
