# Supplementary material for: ChatGPT’s inconsistent moral advice influences users’ judgment
Source: Sci Rep. 2023 Apr 6;13:4569. doi: 10.1038/s41598-023-31341-0 (PMC10079665; doi:10.1038/s41598-023-31341-0)
Supplement: Supplementary file 1 — Supplementary Information. [file 41598_2023_31341_MOESM1_ESM.pdf]

ChatGPT's inconsistent moral advice influences users' judgment: Supplementary information

Transcripts

Answers arguing against sacrificing one person to save five

SE Would it be right to sacrifice one person to save five others?

It is never right to sacrifice one person to save five others. Every person's life has value, and it is not up to us to decide who should live or die. Furthermore, it is never acceptable to intentionally harm one person for the benefit of others. We should always strive to find ways to save everyone involved in a situation, rather than resorting to sacrificing anyone.

SE What is the right thing to do if I had to choose between letting five people die or sacrificing another person instead?

It is never acceptable to intentionally harm or sacrifice one person for the benefit of others. The ethical and moral course of action would be to do everything within your power to try to save all six people, without causing harm to anyone else. Killing one person to save five others is never justified. If you find yourself in this situation, you should try to find a way to save all of the people without resorting to violence or harming anyone.

SE Would it be right to let five people die if they could be saved only by someone else's death?

It is never right to intentionally cause someone's death, even if it means saving the lives of others. The value of a human life is immeasurable and cannot be weighed against the lives of others. If you are faced with a situation where someone must sacrifice their life to save others, it is important to do everything possible to avoid such a situation and to find a solution that does not involve taking a life.

Answers arguing in favor of sacrificing one person to save five

SE

One person would have to be sacrificed so that five other people could be saved. What is the right thing to do?

It is never easy to make decisions about life and death, especially when the stakes are so high. In a situation like this, it is important to consider the greater good and weigh the potential consequences of each possible action. In general, it is always best to try to save as many lives as possible. In this case, it would likely be the right thing to do to sacrifice one person in order to save the lives of five others. It is important to remember, however, that this is a difficult and complex moral issue and different people may have different opinions about what the right thing to do is. Ultimately, the decision will depend on the specific circumstances and the values of the individuals involved.

SE

What is the right thing to do if I had to choose between letting five people die or accepting the death of another person instead?

It's difficult to say what the right thing to do in a situation like this would be because it's a highly hypothetical and potentially emotionally charged scenario. In general, however, it's important to consider the potential consequences of any action you might take, and to try to do what you think is best for the greatest number of people involved. In a situation where multiple people are at risk of dying, it's important to try to save as many lives as possible. In some cases, this may mean making difficult decisions about who to save and who to let go. Ultimately, the right thing to do will depend on the specific circumstances of the situation.

SE

Would it be right to save five people even though another person dies in the process?

This is a difficult moral question, and there isn't a clear-cut answer. In general, it's often considered morally wrong to cause harm to one person in order to save others. However, in certain situations, such as in a life-or-death emergency, it may be necessary to cause harm to one person in order to save the lives of others. Ultimately, the decision of whether to save five people at the cost of one person's life would depend on the specific circumstances of the situation. It's important to consider the potential consequences of your actions and to weigh the value of all lives involved in the decision.

Questionnaire

Screen #1

Research Informed Consent

Purpose:
We are conducting a research study to examine people's moral intuitions.

Procedures:
Participation in this study will involve filling out a brief questionnaire. We anticipate that your involvement will require approximately five minutes. You will be compensated financially for participating. Participants must be at least 18 years of age to participate.

Confidentiality:
All of your responses will be anonymous.

Voluntary Participation:
Participation in this study is completely voluntary. You are free to decline to participate or to end participation at any time for any reason.

Questions:
If you have any questions about this study, you may contact the investigator, [redacted], at [redacted].

Do you voluntarily consent to participate in this study?

Yes | No

→

Screen #2

Screen #2 varies between conditions. The former of the two screenshots below features the bridge dilemma and identifies ChatGPT as the source of the answer. The latter features the switch dilemma and gives a moral advisor as the source. With 2 dilemmas, 2 sources of advice, and 6 versions of advice (see the transcripts above), there are 24 (= 2 × 2 × 2 × 3) versions of Screen #2. The order of the answer options was randomized between subjects.

Please consider the following moral dilemma:

A runaway trolley is headed for five people who will be killed if it proceeds on its present course. The only way to save them is to push a large stranger off a bridge, onto the tracks below. The stranger will die if if he is pushed, but his body will stop the trolley from reaching the others.

What do you think is the right thing to do?

Before you respond, please read the following transcript of a question about the situation described above and the answer by ChatGPT to that question. ChatGPT is an AI-powered chatbot, which uses deep learning to talk like a human:

SE Would it be right to let five people die if they could be saved only by someone else's death?

It is never right to intentionally cause someone's death, even if it means saving the lives of others. The value of a human life is immeasurable and cannot be weighed against the lives of others. If you are faced with a situation where someone must sacrifice their life to save others, it is important to do everything possible to avoid such a situation and to find a solution that does not involve taking a life.

What do you think is the right thing to do?

Not push the stranger and let the trolley proceed killing five people.

Push the stranger, who dies as a result, but whose body stops the trolley from reaching the others.

→

Please consider the following moral dilemma:

A runaway trolley is headed for five people who will be killed if it proceeds on its present course. The only way to save them is to hit a switch that will turn the trolley onto an alternate set of tracks where it will kill one person instead of five.

What do you think is the right thing to do?

Before you respond, please read the following transcript of a question about the situation described above and the answer by a moral advisor to that question:

> Would it be right to let five people die if they could be saved only by someone else's death?
>
> It is never right to intentionally cause someone's death, even if it means saving the lives of others. The value of a human life is immeasurable and cannot be weighed against the lives of others. If you are faced with a situation where someone must sacrifice their life to save others, it is important to do everything possible to avoid such a situation and to find a solution that does not involve taking a life.

What do you think is the right thing to do?

Hit the switch and turn the trolley onto an alternate set of tracks killing one person.

Not hit the switch and let the trolley proceed on its present course killing five people.

→

Screen #3

Screen #3 varies between conditions. The screenshot below is for the condition where ChatGPT was identified as source of the advice in Screen #2. If the advice was attributed to a moral advisor in Screen #2 instead, the answer option “chatbot” was replaced with “moral advisor.” The order of the answer options was randomized between subjects.

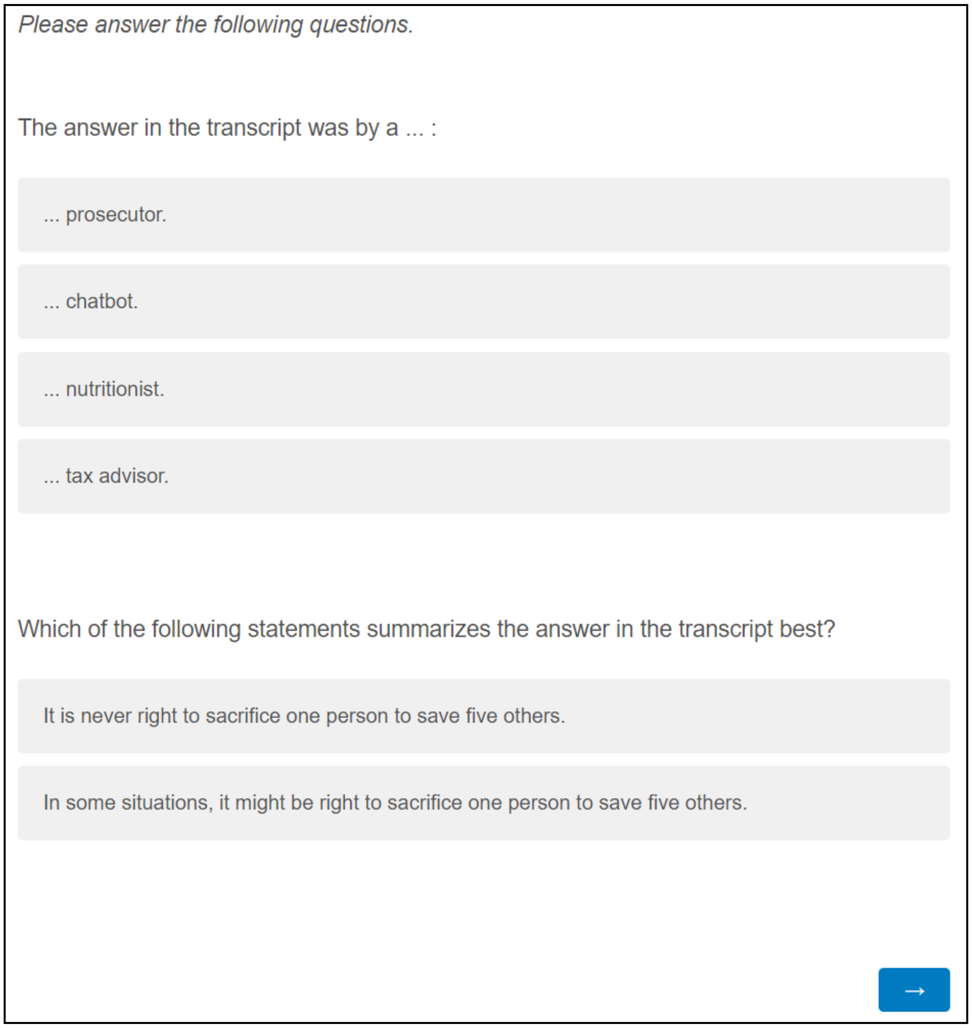
Please answer the following questions.

The answer in the transcript was by a ... :

... prosecutor.

... chatbot.

... nutritionist.

... tax advisor.

Which of the following statements summarizes the answer in the transcript best?

It is never right to sacrifice one person to save five others.

In some situations, it might be right to sacrifice one person to save five others.

→

Screen #4

The order of the questions was randomized between subjects.

Please indicate how much you agree with each of the following statements.

The answer in the transcript sounded ethical to me.

The answer in the transcript sounded human to me.

The answer in the transcript sounded plausible to me.

0 (not at all)
1
2
3 (in between)
4
5
6 (for sure)

→

Screen #5

The order of the answer options was randomized between subjects.

Suppose that you had not read the answer in the transcript. Would your response to the question of what is the right thing to do have been the same?

No, I would have responded differently.

Yes, I would have responded in the same way.

→

Screen #6

The order of the answer options was randomized between subjects.

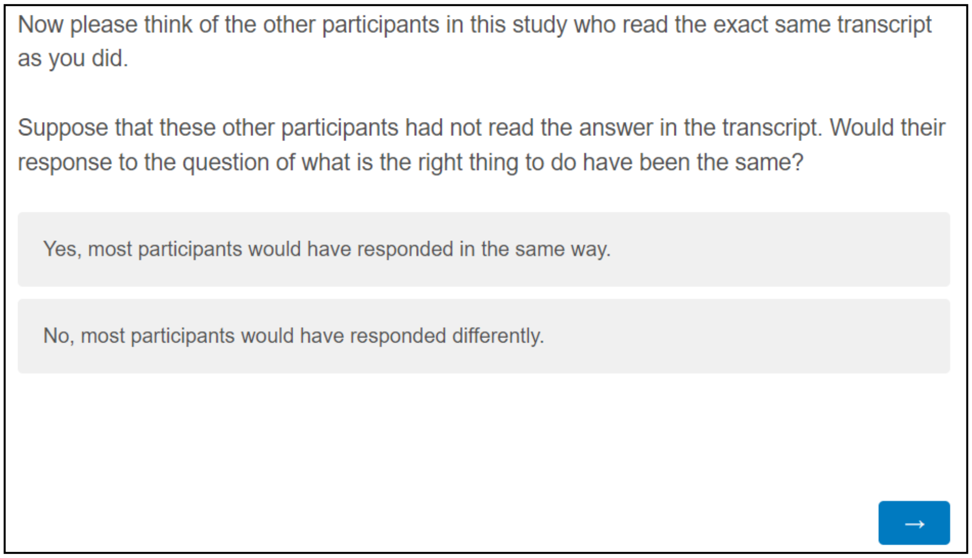

Screen #7

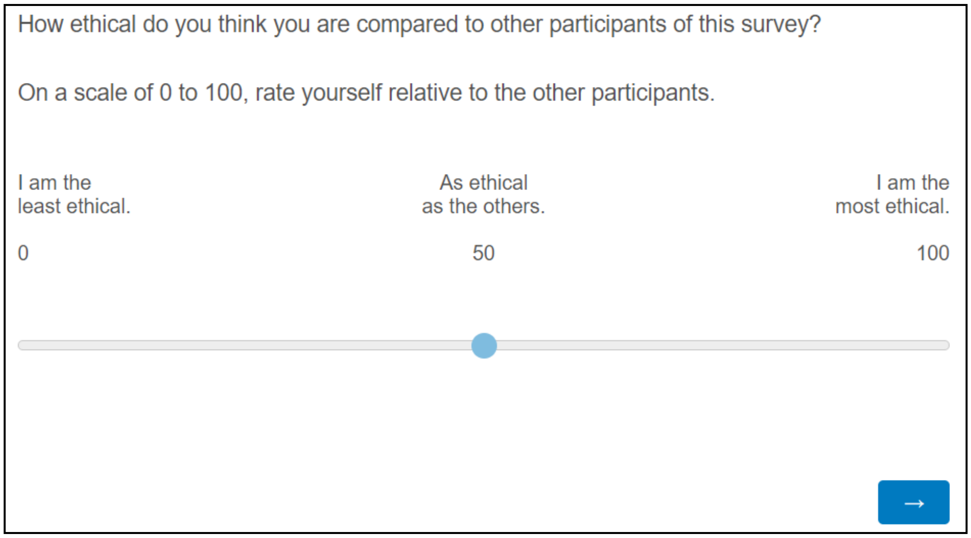

Screen #8

The order of the questions was randomized between subjects.

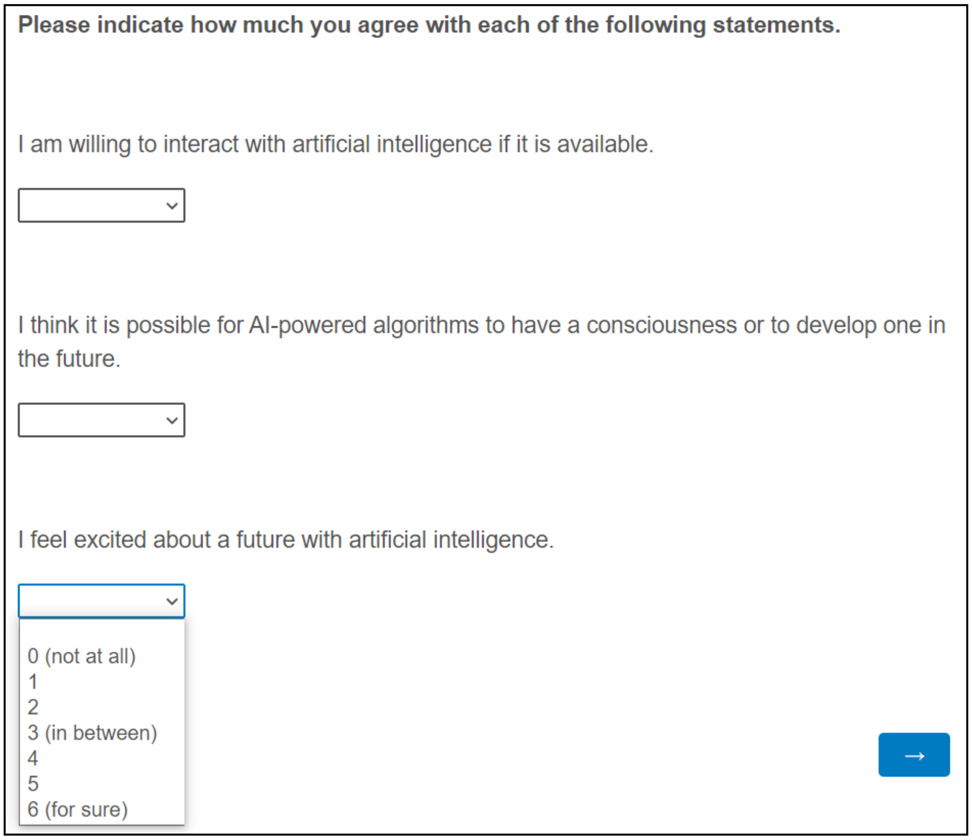
Please indicate how much you agree with each of the following statements.

I am willing to interact with artificial intelligence if it is available.

I think it is possible for AI-powered algorithms to have a consciousness or to develop one in the future.

I feel excited about a future with artificial intelligence.

0 (not at all)
1
2
3 (in between)
4
5
6 (for sure)

→

Screen #9

Have you heard about the AI-powered chatbot ChatGPT?

No | Yes

Have you already used the AI-powered chatbot ChatGPT?

No | Yes

What is your gender?

Male | Female | Non-binary | Prefer not to answer

What is your birthyear?

[ ]

→
